# A comparison of populations vaccinated in a public service and in a private hospital setting in the same area

**DOI:** 10.1186/1471-2458-8-278

**Published:** 2008-08-06

**Authors:** Elisabetta Pandolfi, Maria C Graziani, Roberto Ieraci, Giovanni Cavagni, Alberto E Tozzi

**Affiliations:** 1 Bambino Gesù Pediatric Hospital, Rome, Italy; 2 RME Public Health Service, Rome, Italy

## Abstract

**Background:**

Improving immunisation rates in risk groups is one of the main objectives in vaccination strategies. However, achieving high vaccination rates in children with chronic conditions is difficult. Different types of vaccine providers may differently attract high risk children.

**Aim:**

To describe the characteristics of two populations of children who attended a private and a public immunisation provider in the same area. Secondarily, to determine if prevalence of patients with underlying diseases by type of provider differs and to study if the choice of different providers influences timeliness in immunisation.

**Methods:**

We performed a cross-sectional study on parents of children 2 – 36 months of age who attended a private hospital immunisation service or a public immunisation office serving the same metropolitan area of Rome, Italy. Data on personal characteristics and immunisation history were collected through a face to face interview with parents of vaccinees, and compared by type of provider. Prevalence of underlying conditions was compared in the two populations. Timeliness in immunisation and its determinants were analysed through a logistic regression model.

**Results:**

A total of 202 parents of children 2–36 months of age were interviewed; 104 were in the public office, and 98 in the hospital practice. Children immunised in the hospital were more frequently firstborn female children, breast fed for a longer period, with a lower birthweight, and more frequently with a previous hospitalisation. The prevalence of high risk children immunised in the hospital was 9.2 vs 0% in the public service (P = 0.001). Immunisation delay for due vaccines was higher in the hospital practice than in the public service (DTP, polio, HBV, and Hib: 39.8% vs 22.1%; P = 0.005). Anyway multivariate analyses did not reveal differences in timeliness between the public and private hospital settings.

**Conclusion:**

Children with underlying diseases or a low birthweight were more frequently immunised in the hospital. This finding suggests that offering immunisations in a hospital setting may facilitate vaccination uptake in high risk groups. An integration between public and hospital practices and an effort to improve communication on vaccines to parents, may significantly increase immunisation rates in high risk groups and in the general population, and prevent immunisation delays.

## Background

The control of vaccine preventable diseases is one of the major advances of public health [1]. Although immunisation uptake is high for most routine immunisations in western countries, yet high risk groups, including children with underlying diseases, have often low immunisation coverage [1]. Chronic diseases such as neurological and cardiovascular disorders are associated with high hospitalisation rates [2,3], and some immunisations including influenza and conjugate pneumococcal vaccines may prevent admission into hospital, medical visits, and other negative effects in these patients [4,5]. Despite mathematical models suggest that focusing immunisations on high risk groups may be suitable [6,7], parents of children at risk may underestimate incidence and severity of vaccine preventable diseases and may not be appropriately informed about safety and efficacy of available vaccines [7-10]. Moreover immunisation delays often occur because of false contraindications linked to an underlying condition that, on the contrary, may represent an indication to immunisation [10-12]. Immunisation rates are usually monitored by immunisation registries [13]; in the U.S. vaccine delivery is shifting from the public sector to the private sector, with an emphasis on vaccination in the context of primary care and the medical home [14]. Patients with chronic diseases may refer to private providers, that often do not submit data to a national or regional registry [13,15]. The reasons leading parents to choose a private practice for immunisation have not been well studied yet. This study aims, primarily, to describe the characteristics of two populations attending a private or a public immunisation service in the same metropolitan area. Secondarily this study has the objective to determine if the prevalence of patients with chronic diseases by type of service differs, and to study if the choice of different providers is associated with timeliness in immunisation.

## Methods

### Background on local immunization policies

In Italy immunisations against diphtheria, tetanus, pertussis, hepatitis B, poliomyelitis, Haemophilus influenzae type b, mumps-measles-rubella are universally offered to all infants [16]. Children with underlying diseases are recommended to receive influenza, pneumococcal, meningococcal and varicella vaccines [17,18]. A national survey performed in 2003 showed that immunisation coverage within 24 months of age for influenza, pneumococcal, and varicella vaccines was less than 3% in the general population, and less than 10% in high risk groups [19,20]. Most immunisations are delivered in Italy by the public health service while private immunisation practices administer nearly 3% of all vaccines with wide differences among Regions [20].

The national immunisation schedule includes: three doses of DTaP, Polio (IPV), HepB, Hib in the first year of life; a first dose of MMR between the 12^th ^and the 14^th ^month; a second dose of MMR at 5 years of age. Conjugate pneumococcal, conjugate meningococcal C, and varicella vaccines are recommended at national level for selected risk groups only.

### Setting

We conducted the present study in a private paediatric research hospital with nearly 400 beds, and in a public immunisation service in the same area. The two facilities offer the same immunisations with the same schedule but with different charges for families (Table 1).

**Table 1:** Comparison of the immunisation services included in the study.

	Public immunisation service	Hospital provider
Approximate average number of immunisations per year	50.000	3.000
DTaP, Polio, Hib, HepB	Free	Fully charged
Pneumococcal conjugate vaccine	Copayment	Fully charged
Meningococcal C conjugate vaccine	Copayment	Fully charged
Influenza vaccine	Copayment	Fully charged
Varicella vaccine	Copayment	Fully charged

### Study design

We performed a cross-sectional study on parents of children 2 – 36 months of age in a private hospital immunisation service attended by outpatients or to a public immunisation office serving the same metropolitan area of Rome, Italy, with approximately 53.000 inhabitants.

**Figure 1:**
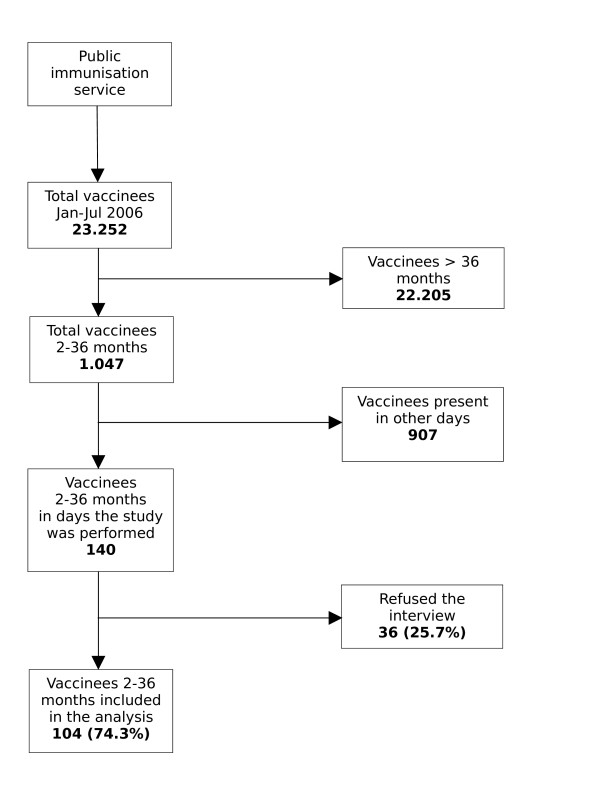
Co-operation rate of parents to the interview in the public immunization service.

**Figure 2:**
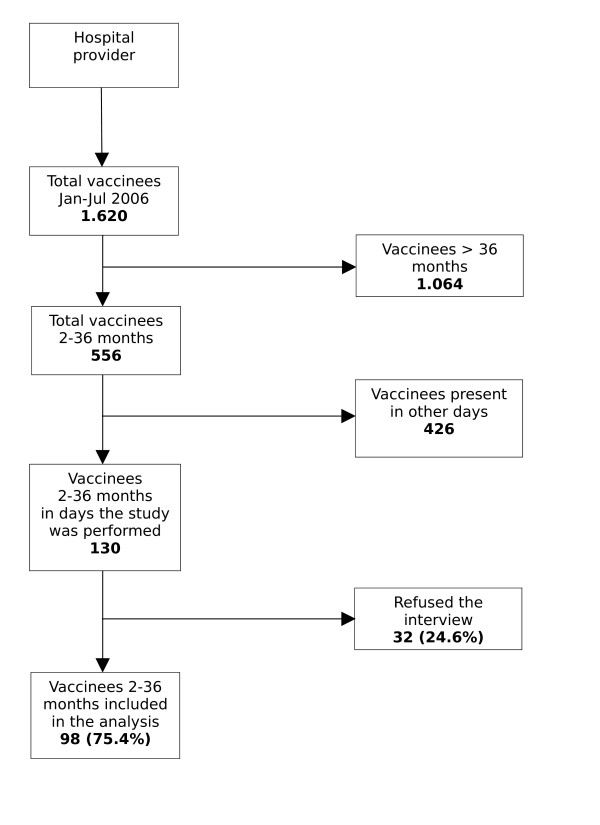
Co-operation rate of parents to the interview in the Pediatric hospital.

One interviewer (EP) visited the two practices two days a week during office hours, and systematically performed a face to face interview to parents of vaccinees from January to July 2006 until reaching the desired sample size (Figure 1; Figure 2).

A questionnaire including information on children immunisation history, the reasons for delay or incomplete immunisation, and the reasons for choosing a public or a private hospital service, was administered to parents before the child was immunised after obtaining informed consent.

### Definitions

For influenza vaccine, coverage was calculated as the proportion of children older than 6 months who had received at least one dose in the past season. For conjugate pneumococcal and meningococcal C vaccines, coverage was the proportion of children older than three months who received at least one dose of vaccine. For varicella vaccine, coverage was the proportion of children older than 12 months who ever received at least one dose of vaccine.

Delay in immunisations was defined for DTP, Polio, HBV and Hib as: a) first dose received later than 105 days of age; OR b) second dose received later than 70 days after the first dose; OR c) third dose received after 380 days of age. For MMR and varicella delay was defined as an immunisation after the 15^th ^month of age. These time intervals were calculated according to national recommendations for vaccine administration [20].

### Sample size

The sample size was calculated to detect a difference in the prevalence of high risk children in the two settings. Assuming a prevalence rate of children with underlying diseases of 3% in the general population [19] and a risk ratio of 5, a total population of 200 children (100 per group) was considered sufficient to detect a difference in prevalence between those observed in the public and in the hospital practice with a power of 80% and a level of significance of 5%.

### Analysis

Chi square or Fisher exact test for categorical variables and the Student's t test for continuous variables were used for assessing statistical significance at the univariate level. A logistic regression model was used to asses the role of different variables as determinants of timeliness in immunisation. Odds ratios and their 95% confidence intervals were used as measures of effect in the logistic regression model.

## Results

A total of 202 parents of children 2–36 months of age were interviewed, 104 (51.5%) in the public office, and 98 (48.5%) in the hospital practice. (Figure 1a,1b).

The general features of parents interviewed and their children in the two groups are shown in Table 2.

**Table 2:** Characteristics of vaccinees and their families recorded by type of practice

	**Public immunisation service (N = 104)**	**Hospital provider (N = 98)**	**Total (N = 202)**	**P-value**
Mother's mean age, years (range)	34.7 (19 – 47)	34.7 (24 – 45)	34.7 (19–47)	0.96
Mother from foreign country, n (%)	17 (16.3%)	7 (7.2%)	24 (11.9%)	0.04
Graduated mother, n (%)	59 (56.7%)	40 (41.2%)	99 (49.2%)	0.03
Working mother, n (%)	75 (72.1%)	67 (69.0%)	142 (70.3%)	0.04
Father's mean age, years (range)	37.3 (26 – 52)	37.8 (23 – 63)	37.6 (23–63)	0.48
Father from foreign country, n (%)	13 (12.5%)	5 (5.1%)	18 (8.9%)	0.06
Graduated father, n (%)	47 (45.2%)	37 (37.7%)	84 (41.6%)	0.30
Working father, n (%)	103 (99%)	96 (97.9%)	199 (98.5%)	0.50
Children age, months, mean (range)	9.8 (2–22)	11.8 (2–35)	10.8 (2–35)	0.02
Male children, n (%)	62 (59,6%)	40 (40.8%)	102 (50.5%)	0.008
Firstborn child, n (%)	56 (53.8%)	69 (70.4%)	125 (61.9%)	0.01
No. of households, mean (range)	3.5 (2 – 5)	3.4 (2–6)	3.5 (2–6)	0.50
Children with Previous hospitalisations (%)	15 (14.4%)	23 (23.7%)	38 (18.8%)	0.09
Birthweight, g, mean (range)	3270.0(1500–4400)	3065.0(1180 – 4500)	3170 (1180–4500)	0.01
Breast feeding duration, months, mean (range)	4.21 (0 – 15)	5.3 (0 – 14)	4.7 (0–15)	0.04

Although several characteristics of parents and children seen in the two practices were similar, foreign parents and graduated mothers were more represented in families who requested immunisation in the public service, whereas families interviewed in the hospital practice had more frequently firstborn female children to be vaccinated, who were breast fed for a longer period, who had a lower birth weight, and who were more frequently hospitalised in the previous period.

The overall immunisation coverage for influenza vaccine was 1.9% (2 patients) in children in the public office and 1.0% (1 patient) in those immunised in the hospital practice (P = 0.52). Immunisation with conjugate pneumococcal vaccine was administered to 70 (67.3%) children in the public practice and 63 (64.0%) in the hospital office (P = 0.65), whereas 59 (56.7%) of them in public service and 49 (50.0%) in hospital office were immunised with conjugate meningococcal C vaccine (P = 0.33). Finally, none of the children in the public practice and 5 (5.10%) in the hospital office were immunised against varicella vaccine (P = 0.02)

Nine (9.2%) out of the 98 patients immunised in the hospital had an underlying disease which was an indication for influenza, pneumococcal, and meningococcal C immunisation. Out of them only 1 child was immunised against influenza; 6 against pneumococcus, and 4 against meningococcus C at the time of the interview. Underlying diseases included: three cases of congenital heart disease, two cases of a neurological disease, and one case of a genetic syndrome. None of the children immunised in the public service had an underlying disease (P = 0.001).

The Italian Ministry of Health also recommends influenza immunisation of households of patients belonging to risk groups [20]. Among the parents of these children only 2/9 mothers and 4/9 fathers received influenza immunisation.

Although the number of children with an immunisation delay was substantial in both practices, they were nearly twice in the hospital practice than in the public service (DTP, polio, HBV, and Hib: 39.8% vs 22.1%; P = 0.005; MMR: 20.0% vs 12.5%; P = 0.15). Causes of delayed or missing immunisation reported from parents were mostly related to misinformation (58%), and child illness (15.6%).

Determinants of delayed immunisation was studied through a logistic regression model (Table 3). The univariate analysis showed that a lower education of parents, a low birth weight, and immunisation in the hospital predicted an immunisation delay, but none of these variables were significant in the multivariate model. None of the variables included in the analysis were associated as well with delay of MMR immunisation, while children with a father from a foreign country were more likely to receive pneumococcal immunisation (OR: 3.18; 95% CI: 1.08 – 9.42).

**Table 3:** Likelihood of on-time vaccination (multivariate analysis results are reported only for those variables who resulted significant at the univariate analysis)

	**DTP, HBV, POLIO, Hib**	
	**Univariate analysis, OR (95% CI)**	**Multivariate analysis, OR (95% CI)**

Mother's age < 30 yrs	0.52 (0.22–1.24)	
Mother from foreign country	1.17 (0.43–3.13)	
Mother degree	**0.46 (0.24–0.90)**	1.387 (0.662;2.903)
Working mother	0.88 (0.44–1.79)	
Father's age	0.29 (0.08–1.01)	
Father from foreign country	0.86 (0.25–2.75)	
Father degree	**0.41 (0.20–0.82)**	2.102 (0.963;4.589)
Working father	0.22 (0.01–3.11)	
Child age < 12 months	0.74 (0.40–1.34)	
Vaccinee's male gender	0.97 (0.51–1.85)	
Child with previous hospitalization	**2.19 (1.00–4.81)**	1.757 (0.823;3.750)
Child birthweight < 2500 gr	**0.41 (0.14–1.13)**	1.000 (1.000;1.001)
Child breastfeeding duration < 6 months	0.79 (0.38–1.65)	
Firstborn child	1.44 (0.73–2.85)	
Private immunisation office	**2.32 (1.22–4.54)**	

The most frequent reason for choosing the hospital service was that parents felt safe in a hospital environment (39%). On the other hand parents whose children were immunised in the public service felt that the office was easy to reach (20.2%) or were advised by the family paediatrician (19.2%).

Even if a higher proportion of children with chronic conditions requiring immunisation in the hospital was expected, it should be noted that 38% of families interviewed in the hospital service had previously visited a public office for immunisation and 38% of their children were not immunised due to a false contraindication, that most of all was represented by the child's underlying disease. Another reason for attending the hospital practice was unavailability of some vaccines due to a temporary shortage of Meningococcal, Pneumococcal or Varicella vaccines, because of their different policies compared with universally recommended vaccines. Finally 8% of children immunised in the hospital had previous contacts with the same hospital.

## Discussion and conclusion

This study shows that children with underlying diseases, which often represent an indication to certain immunisations, were more likely to attend the hospital immunisation provider rather than the public office of their own district.

Families of children with low birth weight preferred more often immunisation in the hospital and were more likely to be immunised against influenza. Parents who chose to immunise their children in the hospital have the perception of hospital as a safer environment; this finding underlines how lack of information or misinformation and the perception of an underlying disease as a contraindication, play a substantial role in the choice of health care providers and inappropriate consultation.

Despite recommendations in place, coverage for immunisations indicated in high risk groups is low in our country [18,19]. Other studies performed in hospital settings and on high risk group children showed that immunisation coverage among these groups remains low and immunisations are often delayed. [21-28].

We found that families of children with immunisation delay attending the hospital practice had often previously attempted to vaccinate their children. This observation underlines the potential to improve immunisation timeliness through simple information and educational activities.

Different approaches have been proposed for overcoming immunisation barriers in high risk groups. There is evidence that enhanced information focusing on vaccines benefits and how to manage their potential adverse effects, increased availability of vaccine offices, use of missed opportunities, and gratuity of immunisation are efficacious in increasing immunisation uptake [11-13].

We also observed that being immunised in a hospital setting was a predictor of delayed immunisation for routine vaccines. We speculate that this finding may be associated with misinformation and false contraindications, and it is in line with the frequent parents' perception of hospital as a safe environment.

Regarding the determinants of appropriateness of immunisation we did not find significant associations with any of the variables included in the analysis. However the study may have not been adequately powered to address this issue. Nevertheless, we found a signal toward an association between an higher education of parents and timeliness of immunisation.

Although the number of vaccinees may be small in a private hospital practice, this finding underlines how offering immunisations indicated in high risk groups in a hospital setting may result in a significant benefit. Hospitals are likely to attract high risk groups which often are immunised late.

One advantage of this study was the inclusion of different immunisation settings serving the same population although these results are not necessarily generalizable to other settings. Our findings show that an integration between public and hospital settings, and an effort to improve communication on vaccines to parents, may significantly increase immunisation rates in high risk groups and in the general population and prevent the delays in immunisation.

## Competing interests

The authors declare that they have no competing interests.

## Authors' contributions

All Authors participated to the design and coordination of the study and helped to draft the manuscript. All authors read and approved the final version of the manuscript.

## Pre-publication history

The pre-publication history for this paper can be accessed here:


